# Molecular detection of multidrug and methicillin resistance in *Staphylococcus aureus* isolated from wild pigeons (*Columba livia*) in South Africa

**DOI:** 10.1016/j.onehlt.2023.100671

**Published:** 2024-01-01

**Authors:** Trevor K. Wilson, Oliver T. Zishiri, Mohamed E. El Zowalaty

**Affiliations:** a Discipline of Genetics, School of Life Sciences, College of Agriculture, Engineering and Science, University of KwaZulu-Natal, Private Bag X54001, Durban 4000, South Africa; b Veterinary Medicine and Food Security Research Group, Medical Laboratory Sciences, Faculty of Health Sciences, Abu Dhabi Women's Campus, Higher Colleges of Technology, Abu Dhabi 41012, United Arab Emirates

**Keywords:** *Staphylococcus aureus*, Antimicrobial resistance, Wild pigeons, *Columba domestica livia*, *Columba livia*, Houseflies, *Musca domestica*, One health, Antibiotic resistance genes, MRSA, *mecA*, methicillin resistance

## Abstract

*Staphylococcus aureus* is an important human and veterinary pathogen. The present study aimed to determine the prevalence of antibiotic resistance among *S. aureus* isolated from samples obtained from free-flying wild pigeons and houseflies from different locations surrounding a local hospital in the Greater Durban area in KwaZulu-Natal Province, South Africa. Environmental fecal samples were obtained from wild pigeons that inhabits the grounds of a local public hospital located on the South Beach area, Durban, South Africa. Housefly samples were collected from three different locations (Kenneth Stainbank Nature Reserve, Montclair/Clairwood, and Glenwood/Berea) in the greater Durban area, all within a close proximity to the hospital. Following enrichment, identification, and antimicrobial resistance profiling, *S. aureus* isolates were subjected to DNA extraction using the boiling method. It was found that 57 out of 252 samples (22.62%) were positive for *S. aureus*. The Kirby-Bauer disk diffusion method of antibiotic susceptibility testing was performed and revealed that antibiotic resistance rates to penicillin and rifampicin were the most common, with both returning 48 (84.2%) out of the 57 *S. aureus* isolates being resistant to penicillin and rifampicin. Antibiotic resistance rates to clindamycin, linezolid, erythromycin, tetracycline, cefoxitin, and ciprofloxacin were 82.5%, 78.9%, 73.7%, 63.2%, 33.3%, and 15.8% respectively. Antibiotic resistance genes were detected using primer-specific PCR and it was found that the prevalence rates of *tetM, aac(6′)–aph(2″), mecA, tetK, ermc*, and *blaZ* genes were 66.7%, 40.4%, 40.4%, 38.6%, 24.6%, and 3.51% respectively. Statistical analysis revealed significant (*p* < 0.05) relationships between the *tetM, aac(6′)–aph(2″),* and *ermC* genes and all parameters tested. A significant correlation between the *aac(6′)–aph(2″)* gene and the *tetM* (0.506) and *ermC* (−0.386) genes was identified. It was found that 23 (40.3%) *S. aureus* isolates were *mecA* positive, of which 10 (52.6%) out of 19 cefoxitin-resistant isolates were *mecA* positive and 13 (35.1%) out of 37 cefoxitin-sensitive isolates were *mecA* positive. The results of the present study demonstrated the detection of methicillin and multidrug resistant *S. aureus* isolated from samples obtained from wild pigeons and houseflies in the surroundings of a local public hospital in the Greater Durban area in South Africa. The findings of the study may account for the emergence of multidrug-resistant staphylococcal infections. The findings highlight the significant role of wild pigeons and houseflies in the spread of drug-resistant pathogenic *S. aureus* including MRSA. The conclusions of the present study highlight the improtant role of wildlife and the environment as interconnected contributors of *One Health*.

## Highlights

• Wild pigeons are reservoirs of plethora of bacteria, viruses, fungi and parasitic pathogens of zoonotic potential. No information is available concerning the isolation and antimicrobial profiling of *S. aureus* or other bacterial pathogens isolated from wild pigeons in South Africa.
• The study is the first in South Africa to report the detection of multidrug resistant *S. aureus* isolated from fecal and feather samples of wild pigeons, and housefly samples.
• *S. aureus* isolates in the present study were alarmingly resistant to commonly used antibiotics representing increased concerns to public health and veterinary health.
• The wild pigeons in the present study originated from flocks inhabiting the surroundings of a local public hospital and are close to human activities in the Greater Durban area which poses public health concerns.
• The conclusions of the present study highlight the significance of wildlife and the environment as interconnected contributors of *One Health*.

## 1 Introduction

Antimicrobial resistance is one of the major and foremost threats to public health globally, where the interconnected transmission and emergence are contributing aspects to its development [1]. While the use of antibiotics in humans significantly contributes to the development and emergence of antibiotic resistance, a number of ecosystems play synergistic roles in the dissemination of antimicrobial resistance across various species [2]. Environmental wastewater, livestock, companion animals, and wildlife are important contributors to the development and emergence of antibiotic resistance [[2], [3], [4]]. Antimicrobial resistance is considered a *One Health* dilemma. The potential causes and interconnected human-animal- environment driving factors which contribute to the spread of antimicrobial resistance in a *One Health* perspective were recently reported and reviewed elsewhere [2,5,6].

Wild birds of different taxa and species are major reservoirs of diverse antimicrobial resistance genetic pools [[7], [8], [9], [10]]. The presence of antimicrobial resistance in wildlife is directly related to the anthropogenic activity pressure on the ecosystems [4,8]. It was previously reported that several wildlife species can be considered sentinels for the environmental pressure of antimicrobial resistance, and wildlife surveillance for antimicrobial resistance should be a priority [11].

Feral pigeons (rock pigeons) (*Columba livia* or *Columba livia forma urbana)*) also called city doves, city pigeons, or street pigeons, are descended from domestic pigeons (*Columba livia domestica* Protonym: *Columba domestica livia*) that have returned to the wild [12]. Wild pigeons (*Columba livia* [wild type] (= *Columba livia*) Family *Columbidae* Order *Columbiformes*) (Protonym: *Columba domestica livia* Family *Columbidae* Order *Columbiformes*) commonly known as rock doves are one of the most common wild birds found globally, with twelve subspecies having been identified [12]. Among bird species, wild pigeons are considered good sentinels for antimicrobial resistance studies and different resistant pathogens have been previously reported in wild pigeons such as *E. coli, Staphylococcus aureus*, *Salmonella*, *Chlamydia, Listeria, Campylobacter*, and *Acinetobacter*, among other species including fungi such as *Cryptococcus* and *Candida*, and parasites as well as different viruses [[13], [14], [15], [16], [17], [18], [19]]. Wild pigeons may be responsible for the zoonotic spread of antimicrobial resistance genes and diverse pathogens due to their wide spectrum flying ability between different locations and their proximity to humans [7,20,21]. Moreover, the ability of wild pigeons to adapt diverse urban habitats and their indoor nesting behaviour altogether contribute to their potential role as a source of infection in other susceptible hosts incluidng humans [22].

Feral pigeons are so widely distributed in large populations and thrive in urban and rural areas in South Africa. Pigeons frequently come in close contact with other hosts including humans, livestock and wild birds in parks, public gardens, temples, and farms. Recently, the increase in wild pigeon populations has raised public health and animal health concerns [23].

There is very limited data regarding the detection of antimicrobial resistance in bacterial pathogens incluidng *Staphylococcus aureus* (*S. aureus*) isolated from wild pigeons in South Africa. *S. aureus* causes staphylococcal food poisoning and is implicated in several difficult-to-treat infections in animals and humans. *S. aureus* is one of the most common pathogens in pigeons [13] and it is included among the ESKAPE (*Enterococcus faecium, Staphylococcus aureus, Klebsiella pneumoniae, Acinetobacter baumannii, Pseudomonas aeruginosa*, and *Enterobacter* spp.) list of high priority pathogens [24,25].

It was previously reported that *S. aureus* was detected in public hospital environments in KwaZulu-Natal Province in South Africa [26]. Recently, we reported the isolation and molecular detection of virulence determinants in *S. aureus* isolated from fecal and feather samples obtained from feral pigeons in KwaZulu-Natal Province , South Africa [27]. The fecal droppings of wild pigeons may act as reservoir of several pathogens which have a relevant impact on human and livestock health and may cause severe diseases and economic losses [28]. We hypothesized that wild pigeons and houseflies could be apparent contributing factors in the transmission of staphylococcal infections where pigeon populations inhabit the grounds and surroundings of public hospitals. In this context, the objective of the present study was to determine the prevalence and antimicrobial resistance profiling of *S. aureus* isolated from samples obtained from wild pigeons and houseflies, and to detect different antimicrobial resistance determinants using PCR methods in these bacterial isolates.

## 2 Materials and methods

### 2.1 Ethical approval

The project was approved by the Animal Research Ethics Committee of the University of KwaZulu-Natal (Reference, AREC 071/017, and AREC 014/018). The field sampling protocols and the research were conducted in full compliance with Section 20 of the Animal Diseases Act of 1984 (Act No 35 of 1984) and were approved by the South African Department of Agriculture, Forestry and Fisheries (DAFF) (Section 20 approval reference number 12/11/1/5).

### 2.2 Sample collection

**Fig. 1:**
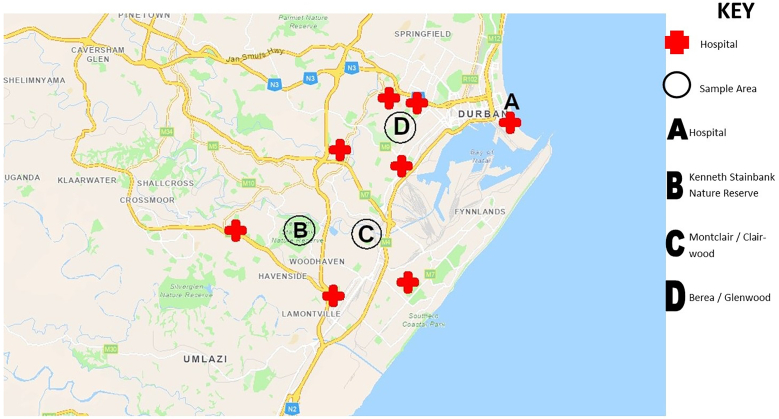
Geographic map showing the locations in the Greater Durban area in KwaZulu-Natal, South Africa where samples in the present study were collected from feral pigeons and houseflies. The map was created using ArcGIS software and was reproduced with permission from [27].

Samples tested in the present study were collected from different locations in the Greater Durban area in South Africa as shown in Fig. 1 as previously reported [27]. In brief, environmental fecal material and feather samples were collected from wild pigeons outside a local public hospital, in the Greater Durban area in KwaZulu-Natal Province during winter (June to August) and spring (September to November) months between 2018 and 2022. Fresh fecal samples were obtained from wild pigeons using sterile Amies agar transport swabs (Thermo Fisher Scientific, Waltham, MA, USA). Free pigeon feathers that appeared fresh and uncontaminated by pigeon feces were collected from the sampling site and were placed in screw cap tubes containing 10 mL of 0.1% peptone water. Whole flies were collected using disposable fly traps which were placed around the sample sites. The captured flies were then placed in tubes containing 70% ethanol. All samples were then transferred to the laboratory for further analysis.

### 2.3 Bacterial isolation

Isolation of *S. aureus* was performed as previously reported [27] by culturing in 0.1% peptone water and brain heart infusion broth followed by isolation on selective media using *S. aureus* ChromoSelect agar supplemented by egg yok tellurite emulsion (Sigma-Aldrich, St. Louis, MO, USA)**.** Environmental fecal and feathers samples collected from wild pigeons, and samples from houseflies were used to isolate presumptive *S. aureus* colonies (brownish black colonies) which were then kept as glycerol stocks for further analysis.

### 2.4 Genomic DNA extraction from *S. aureus*

Genomic DNA was extracted from presumptively-identified *S. aureus* colonies using the standard boiling method as previously reported [26,29] and was used as a template for amplification. In brief, a volume of 400 μL from overnight bacterial culture in Tryptone Soya Broth (Sigma-Aldrich, St. Louis, MO, USA) was placed into a sterile microcentrifuge tube and centrifuged for 15 min at 15000 ×*g*. The supernatant was removed, and the pellet was reconstituted into 200 μL of molecular-grade, nuclease-free water, and was centrifuged for 10 min at 15000 ×*g*. The pellet was reconstituted in 100 μL of TE buffer and it was then boiled at 100 °C for 10 min. After boiling, the samples were centrifuged for 1 min at 15000 ×*g*. The final supernatant was placed in a sterile microfuge tube and DNA concentration was measured using Nanorop as previously reported [27].

### 2.5 PCR confirmation of *S. aureus*

Presumptively identified *S. aureus* isolates were confirmed using species-specific thermonuclease (*nuc)* gene PCR as previously reported [30]. Primers (Table 1) were purchased from Inqaba Biotech (Pretoria, South Africa) and successful PCR amplification of the *nuc* gene confirmed the presence of *S. aureus*. The PCR consisted of 12.5 μL 2× DreamTaq Green PCR master mix (Thermo Fisher Scientific, Waltham, MA,), 4 μL template DNA, 6.5 μL nuclease-free water, and 1 μL each of the forward and reverse primers (20 μM primer concentration), making the total volume in the tube 25 μL as previously reported [27]. PCR amplicons were visualized using 1.5% agarose gel electrophoresis using the molecular weight marker 100 bp DNA ladder (Thermo Fisher Scientific, Waltham, MA, USA). The molecular weight marker used was the 100 bp DNA ladder.

**Table 1:** Staphylococcal gene specific-primers used for polymerase chain reaction for the identification and detection of antibiotic resistance determinants of *S. aureus* in the present study.

**Target gene**	**Primer name**	**Sequence (5′-3′)**	**Amplicon Size (bp)**	**Reference**
*nuc*	Primer 1	GCGATTGATGGTGATACGGTT	270	[30]
	Primer 2	AGCCAAGCCTTGACGAACTAAAGC		
*mecA*_LGA251_	*mecA*_LGA251_ MultiFP	GAAAAAAAGGCTTAGAACGCCTC	138	[32]
	*mecA*_LGA251_ MultiRP	GAAGATCTTTTCCGTTTTCAGC		
*tetK*	Primer K1	CAGCAGATCCTACTCCTT	168	[33]
	Primer K2	TCGATAGGAACAGCAGTA		
*tetM*	Primer M1	GTGGACAAAGGTACAACGAG	405	[33]
	Primer M2	CGGTAAAGTTCGTCACACAC		
*ermC*	Primer F	GCTAATATTGTTTAAATCGTCAATTCC	572	[34]
	Primer R	GGATCAGGAAAAGGACATTTTAC		
*blaZ*	Primer 487	TAAGAGATTTGCCTATGCTT	377	[35]
	Primer 373	TTAAAGTCTTACCGAAAGCAG		
*aac (*6′*) – aph (2*′′*)*	aacA-aphD 1	TAATCCAAGAGCAATAAGGGC	227	[36]
	aacA-aphD 2	GCCACACTATCATAACCACTA		

### 2.6 Antimicrobial susceptibility testing

Antimicrobial susceptibility profiling was performed using the Kirby-Bauer disk diffusion method according to the Clinical Laboratory Standards Institute (CLSI) guidelines [31]. An inoculum of *S. aureus* isolate (0.5 McFarland standard) was added onto the surface of Mueller-Hinton agar plate (Oxoid, England), and the inoculum was allowed to dry. Antibiotic discs were placed onto the surface of the agar plates using sterile forceps. *S. aureus* isolates were tested for their susceptibility against ciprofloxacin (5 μg), rifampicin (5 μg), clindamycin (2 μg), linezolid (30 μg), tetracycline (30 μg), penicillin (10 units), cefoxitin (30 μg), and erythromycin (15 μg). The plates were incubated for 24 h and results were reported as the zone of inhibition (nearest whole mm) and were interpreted according to the CLSI breakpoints as previously reported [31].

### 2.7 Molecular detection of antimicrobial resistance genes

Antibiotic resistance genes for methicillin, tetracycline, erythromycin, β-lactamase, and aminoglycosides (*mecA, tetK* and *tetM, ermC, blaZ,* and *aac* (6′) – *aph* (2″), respectively were detected by PCR using genomic DNA as the template using specific primers as shown in (Table 1). PCR products were analyzed by electrophoresis using 1.5% agarose gels.

### 2.8 Statistical analysis

Pearson's Chi-Square and Fisher's Exact tests were performed using the software program SPSS version 28 (IBM SPSS Statistics) to determine significant relationship between the sample type, season of collection, and the resistance genes. A relationship was considered significant if the *p* value was <0.05. Association between the presence/absence of one gene to another was assessed via Pearson's Correlation.

## 3 Results

In the present study, we tested 57 *nuc* gene PCR-positive *S. aureus* isolates for their antibiotic susceptibility profiling and the isolates were screened for the presence of different antibiotic resistance genes using staphylococcal primer-specific PCR (Table 1). The 57 (22.62%) *S. aureus* isolates were obtained from 150 samples from wild pigeons (88 fecal samples and 62 feather samples) and 102 housefly samples as was recently reported [27].

Of the 57 *S. aureus* isolates, 48 (84.2%) isolates were resistant to penicillin, 36 (63.2%) isolates were resistant to tetracycline, 42 (73.7%) isolates were resistant to erythromycin, 45 (78.9%) isolates were resistant to linezolid, 9 (15.8%) isolates were resistant to ciprofloxacin, 47 (82.5%) isolates were resistant to clindamycin, 19 (33.3%) isolates were resistant to cefoxitin, and 48 (84.2%) isolates were resistant to rifampicin as shown in Table 2.

**Table 2:** Antibiotic Susceptibility results of *S. aureus* isolates tested in the present study.

**Antibiotic**	Susceptible*	Intermediate*	Resistant*
Penicillin	9	0	48
Tetracycline	16	5	36
Erythromycin	10	5	42
Linezolid	12	0	45
Ciprofloxacin	42	6	9
Clindamycin	8	2	47
Cefoxitin	37	1	19
Rifampicin	8	1	48

**Fig. 2:**
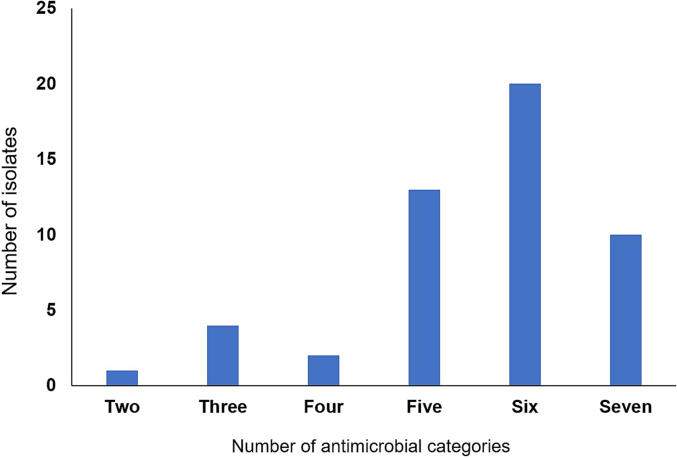
Multidrug resistance of *S. aureus* in the present study. The figure shows the number of *S. aureus* isolates resistant to two, three, four, five, six and seven antimicrobial categories. It was found that 49 out of 57 isolates were resistant to three or more antimicrobial categories and were defined as multidrug resistant *S. aureus* as previously reported [37].

Concerningly, 50 out of the 57 *S. aureus* isolates were resistant to two or more different antibiotic agents. One isolate was resistant to two antibiotic agents, four isolates were resistant to three antibiotic agents, two isolates were resistant to four antibiotic agents, 10 isolates were resistant to five antibiotic agents, 14 isolates were resistant to six antibiotic agents, 13 isolates were resistant to seven antibiotics, and six isolates were resistant to eight antibiotics tested. It was found that 49 isolates were resistant to three or more antimicrobial categories (classes) and were defined as multidrug resistant pathogens as previously reported [37]. One isolate was resistant to two antimicrobial categories, four isolates were resistant to three antimicrobial categories, two isolates were resistant to four antimicrobial categories, 13 isolates were resistant to five antimicrobial categories, 20 isolates were resistant to six antimicrobial categories, 10 isolates were resistant to seven antimicrobial categories as shown in Fig. 2.

The 57 *S. aureus* isolates were screened for different antimicrobial resistance determinants using primer-specific PCR. It was found that out of the 57 tested isolates, 23 (40.4%) isolates were positive for the *mecA* gene. The most prevalent resistance gene was the *tetM* gene with 38 (66.7%) positive isolates, followed by the *aac (‘6′) – aph (2″)* gene with 23 (40.4%) positive isolates, and the *tetK* gene with 22 (38.6%) positive isolates.

In the present study, it was found that the antibiotic resistance gene with the lowest prevalence rate was the *blaZ* gene, with only two (3.5%) isolates were positive, followed by 14 (24.6%) isolates for the *ermC* gene. It was found that 42 (73.7%) isolates out of the 57 *S. aureus* isolates were positive for two or more antibiotic resistance genes, eight (14%) isolates were positive only for one antibiotic resistance gene and none of the tested six antibiotics genes were detected in seven (12.3%) *S. aureus* isolates in the present study. None of the isolates were positive for five or six resistance genes.

**Fig. 3:**
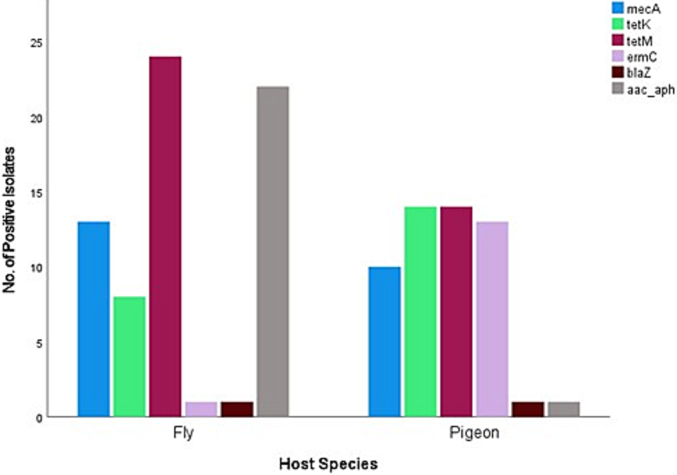
Different antimicrobial resistance genes identified in *Staphylococcus aureus* isolated from wild pigeons and houseflies in the present study. The figure shows that the *ermC* gene was detected almost exclusively in samples obtained from wild pigeons, while the *aac (6’) – aph (2’’)* gene was almost exclusively found in housefly samples.

**Fig. 4:**
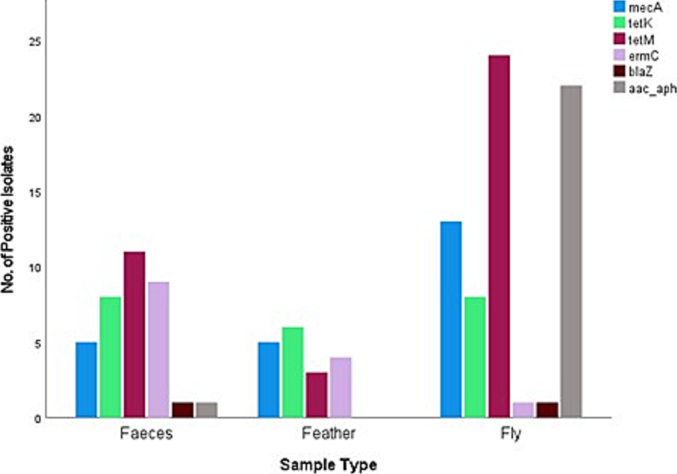
Different antimicrobial resistance genes identified in *S. aureus* isolated from different sample types of wild pigeons and houseflies in the present study. The figure shows that the *aac (6’) – aph (2’’)* gene was detected more frequenty in housefly samples than in pigeon faecal samples, and was not detected in pigeon feather samples.

**Fig. 5:**
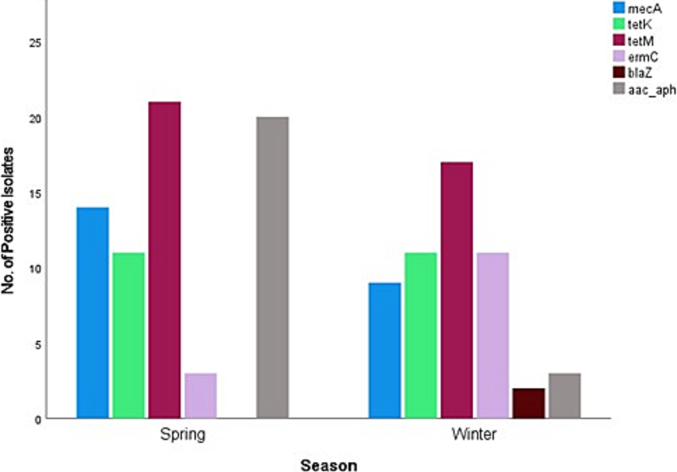
Different antimicrobial resistance genes identified in *S. aureus* isolated from wild pigeons and houseflies in different seasons (winter and spring) in the present study. The figure shows that *ermC* gene was found more frequently in samples collected in the winter than in samples collected in the spring, and that the *aac (6’) – aph (2’’)* gene was found more commonly in samples collected in the spring than in samples collected in the winter.

**Fig. 6:**
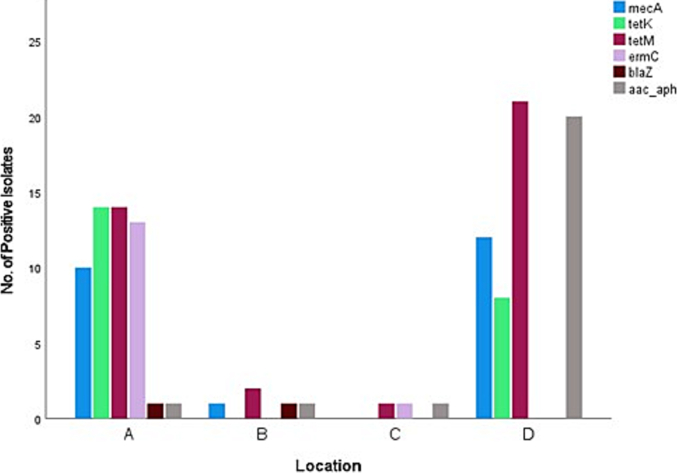
Different antimicrobial resistance genes identified in *S. aureus* isolated from wild pigeons and houseflies from four different locations in the Greater Durban area in KwaZulu-Natal Province in the present study. The figure shows different distribution of the detected resistance genes in the samples collected from different locations. Among the sampling locations, location D appeared to have a greater number of isolates carrying antimicrobial resistance genes than the other locations.

Fig. 3, Fig. 4, Fig. 5, Fig. 6 show the number of *S. aureus* isolates positive for each antibiotic resistance gene from different host species (Fig. 3), sample type (Fig. 4), season of sample collection (Fig. 5), and location of sample collection (Fig. 6). It was found that 21 out of 57 *S. aureus* isolates were positive for two resistance genes, 12 isolates were positive for three resistance genes, nine isolates were positive for four resistance genes, and none of the 57 *S. aureus* isolates were positive for all six resistance genes tested.

Pearson's Chi Square and Fisher's Exact tests were performed to determine if there was a statistically significant (*p* < 0.05) relationship between the sample type, season of sample collection, location of sample collection, and host species and whether the different antimicrobial resistance genes were present (Table 3). Analysis revealed that there is a statistically significant relationship (*p* < 0.05) between the sample type and *tetM*, *ermC*, and *aac (6′) – aph (3″)* antimicrobial resistance genes, as well as these three antimicrobial resistance genes and the location of sample collection and the host species. Analysis also revealed a statistically significant relationship between the season of sample collection and *ermC* and *aac (6′) – aph (2″)* antimicrobial resistance genes. Analysis showed significant correlations between *aac (6′) – aph (2″)* gene and the *tetM* gene (0.506) and the *ermC* gene (−0.386), but no other significant correlations were found (Table 4).

**Table 3:** Probability values for Fisher's Exact and Pearson's Chi-Square tests used to test the relationship between the detected antimicrobial resistance genes and the sample type, season, location, and host species.

**Antibiotic Resistance Gene**	**Sample Type**		**Season**		**Location**		**Host Species**	
	***P*-Values**							
	**Pearson's Chi Square**	**Fisher's Exact**	**Pearson's Chi Square**	**Fisher's Exact**	**Pearson's Chi Square**	**Fisher's Exact**	**Pearson's Chi Square**	**Fisher's Exact**
*mecA*	0.624	0.624	0.117	0.117	0.142	0.118	0.138	0.138
*tetK*	0.498	0.498	0.566	0.566	0.311	0.425	0.201	0.201
*tetM*	<0.001⁎	<0.001⁎	0.151	0.151	<0.001⁎	<0.001⁎	<0.001⁎	<0.001⁎
*ermC*	0.002⁎	<0.001⁎	0.018⁎	0.018⁎	0.004⁎	<0.001⁎	<0.001⁎	<0.001⁎
*blaZ*	1	1	0.254	0.254	0.071	0.071	0.709	0.709
*aac-aph*	<0.001⁎	<0.001⁎	<0.001⁎	<0.001⁎	<0.001⁎	<0.001⁎	<0.001⁎	<0.001⁎

⁎ Figure is significant at the 0.05 level.

**Table 4:** Pearson's Correlation values used to determine the association between antibiotic resistance genes, *p*-values were shown in brackets.

	**Correlations**					
	***mecA***	***tetK***	***tetM***	***ermC***	***blaZ***	***aac_aph***
***mecA***	1	0.156 (0.247)	0.126 (0.349)	−0.054 (0.690)	0.038 (0.782)	0.125 (0.353)
***tetK***	0.156 (0.247)	1	0.025 (0.851)	0.217 (0.104)	−0.151 (0.262)	0.009 (0.947)
***tetM***	0.126 (0.349)	0.025 (0.851)	1	−0.202 (0.132)	0.135 (0.317)	0.506⁎ (<0.001)
***ermC***	−0.054 (0.690)	0.217 (0.104)	−0.202 (0.132)	1	0.113 (0.404)	−0.386⁎ (0.003)
***blaZ***	0.038 (0.782)	−0.151 (0.262)	0.135 (0.317)	0.113 (0.404)	1	0.038 (0.782)
***aac_aph***	0.125 (0.353)	0.009 (0.947)	0.506⁎ (<0.001)	−0.386⁎ (0.003)	0.038 (0.782)	1

⁎ Correlation is significant at the 0.05 level (2-tailed).

## 4 Discussion

*S. aureus* is among the most common pathogenic bacteria found in animals and humans. *S. aureus* produces a variety of extracellular virulence factors and proteases resulting in several infections globally such as infective endocarditis, bacteraemia, respiratory tract, urinary tract, food poisoning, and wound infections [27,38]. The prevalence of this bacterium in recent years can be explained by the difficulty in treating staphylococcal infections due to its escalating resistance levels to commonly used antibiotic agents. Furthermore, the inevitable emergence of resistant strains and multidrug resistance (MDR) is alarming, which is mainly due to the inappropriate use of antibiotics in healthcare and agricultural sectors such as livestock growth promoters and for prophylaxis [39]. The increasing trends in MDR in *S. aureus* isolates was reported from South Africa [26,40,41] and other countries [[42], [43], [44], [45]].

In the current study, we determined the antibiotic resistance profiling and investigated six antibiotic resistance genes using PCR in 57 *S. aureus* which were isolated from 252 samples obtained from wild pigeons and houseflies in the Greater Durban area in KwaZulu-Natal Province, South Africa as previosuly reported [27].

In the present study, the prevalence rate (22.62%) of *S. aureus* obtained from wild pigeons and housefly samples was higher than the prevalence rate of 12.7% of *S. aureus* isolated from environmental samples collected from different frequently touched sites in public hospitals in KwaZulu-Natal province [26]. The prevalence rate in the present study was lower than prevalence rates of 53.6% and 53.8% from commercial broiler chicken and livestock samples, respectively as previously reported [40,41]. This may be explained by differences in sample types, hosts, location and time of sample collection between these studies in KwaZulu-Natal, South Africa.

Different prevalence rates of *S. aureus* were reported from other countries, such as in a study from Iran, a prevalence rate of 6.61% in raw milk samples collected from different shopping centres was reported [43]. A rate of 85% prevalence was reported from samples collected from two hospitals in Egypt [44]. Furthermore, different prevalence rates of 94%, 57.8%, 53.3%, 43.4%, 27.8%, 27.3%, and 3.9%, were reported from the UK, Greece, Brazil, New Zealand, USA, Japan, and China, respectively [[45], [46], [47], [48], [49], [50], [51], [52]].

Altogether, these studies demonstrate the widespread distribution of *S. aureus* in humans, livestock, and ubiquitous environments. In the present study, the detection of *S. aureus* in wild pigeons and houseflies in the surrounding environment of public hospitals is alarming.

The possibility of transmission of pigeon-originated *S. aureus* due to contamination of hospital surroundings from droppings of pigeons via fomites, and mechanical housefly vectors require close monitoring of hygienic practices within public hospitals and the surroundings as well as routine cleaning measures. It was reported that livestock fecal material with antimicrobial resistant pathogens can reach a wider environment via food, drinking water contaminated by pigeon droppings, nesting materials, or even dead carcasses or through the air, run off and aerosolization with the possibility of airborne transmission to other susceptible hosts [[53], [54], [55], [56], [57], [58]].

Furthermore, pigeons are identified as a reservoir of airborne pathogens which represent a health hazard and may be associated with exposure to airborne microorganisms and lead to airborne transmission when pigeons are in close proximity to humans, and other animal hosts. It was previously reported that pathogens of public health and veterinary health significance such as *Escherichia coli, Enterococcus faecium, Clostridium* spp.*, Salmonella* sp.*,* and *Staphylococcus aureus* were detected in the air in a Danish pigeon house [59,60].

*Staphylococcus aureus* is a risk Group 2 human pathogen which in livestock farms [41,59,61] can be present in an antibiotic-resistant variant known as methicillin-resistant *S. aureus* (MRSA), a highly resistant pathogen.

Alarmingly, in the present study, it was found that 49 *S. aureus* isolates were phenotypically resistant to three or more antimicrobial categories and were defined as multidrug resistant pathogens as previously reported [37]. Furthermore, it was found that 42 (73.7%) isolates out of the 57 *S. aureus* isolates were positive for two (n = 21), three (n = 12) or four (n = 9) antibiotic resistance genes, 8 (14%) isolates were positive only for one antibiotic resistance gene and none of the tested six antibiotics genes were detected in 7 (12.3%) *S. aureus* isolates in the present study.

In the present study, the 57 *S. aureus* isolates were highly resistant to penicillin and rifampicin with a rate of 84.2%, and these results concur with a previous study from KwaZulu-Natal Province [41]. The current findings were compared with those of Mkhize et al. [26], and an increase in the resistance rates of the tested antibiotics except for ciprofloxacin was observed for the isolates tested. However, the antibiotic susceptibility trends of *S. aureus* in the present study were different from data reported from other countries [62,63], most likely explained by differences in patterns of antibiotic usage in each country and sample hosts, types, and time of collection.

The escalating antimicrobial resistance levels detected in *S. aureus* is concerning and could result in difficult-to-treat infection [64]. The prevalence of β-lactam, tetracycline, erythromycin, and methicillin resistance genes in *S. aureus* isolates collected from different locations in KwaZulu-Natal Province was previously reported [26,40,41]. The detection of virulence factors and antibiotic resistance in *Staphylococci* isolated from pigeons was reported in other countries [[65], [66], [67], [68], [69], [70], [71]]. Few studies reported the detection of parasitic diseases [72], pigeon paramyxoviruses (Newcastle disease virus) [73], and opportunistic fungi [74] in feral pigeons in South Africa. To the authors' knowledge, no information is available related to the isolation and antimicrobial susceptibility of *S. aureus* and other bacterial pathogens isolated from wild pigeons in South Africa. Nonetheless, very few data is availabe on viral agents in wild pigeons in South Africa.

In the present study, the phenotypic resistance to penicillin, tetracycline, and erythromycin was supported by the molecular detection of *blaZ, tetK/tetM*, and *ermC* resistance genes, which is similar to previously studies which reported *blaZ, mecA, aacA-aphD, ermB, ermC, tetK, tetL,* and *tetM* resistance genes in antibiotic-resistant *S. aureus* isolates [[75], [76], [77]].

Methicillin-resistant *S. aureus* (MRSA) is a notorious pathogen, potential superbug and a major threat to hospitalized patients and the community [32]. In the present study, ten (52.6%) out of the 19 cefoxitin-resistant *S. aureus* isolates were *mecA* gene positive. In contrast, higher *mecA* gene occurrence rates of 82.7% and 100% of cefoxitin-resistant MRSA isolates were previously reported in China and Nigeria [78,79], and a lower *mecA* gene occurrence (5.5%) was previously reported in MRSA in Egypt [80].

In the present study, nine (47.4%) out of the 19 cefoxitin-resistant *S. aureus* isolates did not harbour *mecA* gene similar to a finding reported elsewhere [81]. The lack of *mecA* detection in cefoxitin-resistant *S. aureus* isolates in the present study requires further investigations, however it may be explained by possible existing resistance mechanisms such as *mecA* gene variant, hyperproduction of β-lactamases, and altered affinity of penicillin-binding protein [82].

Interestingly, it was found that 13 (56.5%) out of 23 *mecA* positive *S. aureus* were cefoxitin-sensitive, in other words 13 (35.1%) out of 37 cefoxitin-sensitive isolates were *mecA* positive in the present study. Such cryptic resistance among cefoxitin and oxacillin susceptible *mecA*-positive *S. aureus* was referred to as “stealth” MRSA [83] and was previously reported in strains isolated from patients and food worldwide [[84], [85], [86], [87]].

The finding of *mecA* positive *S. aureus* isolates in the present study requires further investigation and genomic characterization because the underlying mechanisms for this “stealth” MRSA may be complicated and several factors are involved in the resistance to methicillin-like antibiotics in MRSA [83].

The findings of the present study showed low prevalence rates of *ermC* and *blaZ* genes, encoding resistance to erythromycin and β-lactams, respectively. However, the results of the present study showed high prevalence rates of *aac (6′) – aph (2″)* gene encoding aminoglycoside resistance. The results of the current study differ from those reported in China, where the prevalence rates of β-lactams resistance genes were higher, but the prevalence rates of other tested genes were lower than the current results [88]. The antibiotic resistance rates of *S. aureus* isolated from pigeons in the present study were higher than resistance rates of *S. aureus* isolated from pigeons reported from Italy [70]. In contrast, the detection of different bacterial pathogens of public health importance isolated from pigeons in Egypt and their antibiotic susceptibility testing were reported and it was found that the prevalence rates of *S. aureus* isolated from pigeon samples were lower than those in the present study [89].

In the present study, 49 out of 57 *S. aureus* isolates displayed multidrug-resistant phenotype and *mecA* gene was detected in 23 isolates. In a study from Poland, high diversity of *Staphylococcus* species except *S. aureus* was reported and coagulase-negative staphylococci were predominantly isolated from pigeon samples [45]. Contrary to the findings of the present study, none of *Staphylococcus* isolates reported in the Polish pigeon study displayed a multidrug-resistant phenotype and *mecA* gene was not detected in the examined strains and no methicillin-resistant staphylococci were found in pigeons [45]. In contrast, all *S. aureus* strains isolated from fecal samples obtained from pigeons in Brazil were susceptible to the tested antibiotics [69]. The detection of MDR phenotype in *S. aureus* isolates in the present study was consistent with a study from Poland which reported methicillin resistance *Staphylococcus* strains isolated from pigeons [90]. Also, a methicillin-resistant *S. aureus* strain was isolated from a pigeon co-infected with pigeon pox virus [13] and MRSA isolates were detected in the air in pigeon exhibitions in Denmark [71]. A number of pigeon samples were tested and were found to be coinfected with other bacterial and viral pathogens which are beyond the scope of the present study and will be reported elsewhere (Unpublished data, manuscripts in preparation, El Zowalaty, M.E.). The differences in resistance trends between the present study and other studies elsewhere may be explained by the perplexing nature of antimicrobial resistance and multiple contributing factors such as different host species, sample types, time of sample collection, and practices and levels of antibiotic use in the different countries.

In the present study, statistical analysis showed that *mecA*, *tetK*, and *blaZ* genes had no statistically significant (*p* > 0.05) relationship with any variables assessed, but *tetM*, *ermC*, and *aac (6′) – aph (3″)* had statistically significant (*p* < 0.05) relationships to the sample type, location, and host species, and *ermC* and *aac (6′) – aph (3″)* genes had significant relationships to the season of sample collection.

Analysis also revealed that *tetM* gene detected in *S. aureus* isolated from housefly samples was significantly (p < 0.05) greater than the same gene found in *S. aureus* isolated from pigeon samples. The same finding was observed for *ermC* and *aac (6′) – aph (2″)* genes, and for *ermC* gene, a significantly greater number of positive isolates were detected in winter months (June, July, and August), and for *aac (6′) – aph (2″)* gene, a significantly greater number of positive isolates came from the spring months (September, October, and November).

Correlation analysis revealed that *aac (6′) – aph (2″)* gene was found to be significantly correlated to two other genes, *tetM* and *ermC* genes. There was a medium strength, positive correlation with *tetM* gene, meaning that if one of these genes was present, there was a significant chance of the other gene also being present, whereas with *ermC* gene, there was a medium strength negative correlation, meaning that if the gene was present there was a significant chance the other gene would not be present. This finding is similar to previous study by Mkhize et al. [26] which also reproted a positive correlation between *aac (6′) – aph (2″)* and *tetM* genes, but a positive, and not negative, correlation between *ermC* gene and *aac (6′) – aph (2″)* genes. This may be explained by the increase in *aac (6′) – aph (2″)* gene prevalence and decrease in *ermC* prevalence, which most likely influenced the results of the correlation analysis.

In conclusion, we report the detection of methicillin and multiple antibiotic resistant *S. aureus* in fecal droppings and feather samples obtained from free-flying wild pigeons in the vicinity of local public hospitals for the first time in South Africa. The wild pigeons included in the present study came from flocks inhabiting zones close to human activities; therefore, there is a possibility of contact between the pigeons and humans, which may result in zoonotic antimicrobial resistance and possibly zoonotic infections. *S. aureus* isolates in the present study were alarmingly resistant to commonly used antibiotics which represents an increased concern to public health and veterinary health concerning the possible transmission of *S. aureus* and other harbored viral, bacterial, fungal or parasitic agents to other susceptible hosts including human, animal, and poultry. Altogether, the findings of the present study highlight the significance of close monitoring of antimicrobial resistant *S. aureus* and surveillance for pathogens in wildlife. The findings of the present study demonstrated that the emergence and spread of methicillin-resistant *S. aureus* in wild pigeons in South Africa requires a special attention nationally and worldwide. The presented data strongly suggest and recommend the implementation of future studies to identify other bacterial species, viruses, fungal and parasitic pathogens, in addition to multidrug-resistant bacteria in order to delineate and explore the potential role of such birds in the dissemination of infectious diseases and to counteract future pandemics pigeons may be implicated in via zoonotic spillover events. The detection of methicillin and multidrug resistance in *S. aureus* isolated from wild pigeons in South Africa are of grave public health and veterinary health concerns since the pigeon-originated antibiotic resistance genes can potentially spread further to other hosts, local and global communities. Therefore, implementing molecular and genomic surveillance programs are significant for early detection of zoonotic transfer of antibiotic resistance genes. Additionally, strengthening infection prevention and control measures in urban public areas and the surrounding environment of hospitals, particularly on surfaces and areas where pigeons can access is crucial. Furthermore, the conclusions of the present study highlight the significance of wildlife and the environment as interconnected contributors of *One Health*.

## Funding

This work was supported by the South African 10.13039/501100001321National Research Foundation through the Thuthuka Funding Instrument (grant number TTK170411226583).

## CRediT authorship contribution statement

**Trevor K. Wilson:** Methodology, Formal analysis, Visualization, Data curation, Investigation, Validation, Writing – original draft, Writing – review & editing. **Oliver T. Zishiri:** Conceptualization, Methodology, Formal analysis, Investigation, Data curation, Supervision, Funding acquisition, Project administration, Resources, Software, Validation, Visualization, Writing – original draft, Writing – review & editing. **Mohamed E. El Zowalaty:** Conceptualization, Methodology, Investigation, Data curation, Formal analysis, Investigation, Visualization, Validation, Writing – original draft, Writing – review & editing, Project administration, Supervision, Funding acquisition.

## Conflicts of interest

The authors declare that they have no known competing financial interests or personal relationships that could have appeared to influence the work reported in this manuscript.

## Data Availability

Data are available upon reasonable request.
